# Microbiota-gut-brain axis in avian parenting: gut microbiome associates with nest-construction behavior and neural gene expression in a songbird

**DOI:** 10.1186/s42523-025-00486-w

**Published:** 2025-11-18

**Authors:** Cheng-Yu Chen, Hao-Chih Kuo, Yi-Ting Fang, Chia-Wei Lu, Shih-Kuo Chen, Chih-Ming Hung

**Affiliations:** 1 Department of Life Science, National Taiwan University, Taipei, 10617 Taiwan; 2 Biodiversity Research Center, Academia Sinica, Taipei, 11529 Taiwan; 3 Animal Behavior Graduate Group, University of California, Davis, CA 95616 USA

**Keywords:** Nest-construction, Microbiota-gut-brain axis, Zebra finch, Parenting behavior, Lactobacillaceae, Campylobacteraceae

## Abstract

**Background:**

The gut-brain axis mediates bidirectional communication between gut and central nervous activities with gut microbiota acting as a key mediator. While recent studies have mainly focused on how gut microbiota influence non-parenting social behavior in rodents, the role of gut microbiota in parenting behavior, especially in non-model species, is largely unexplored. Nest-building behavior is an early parenting behavior critical to avian survival and evolution, offering an ideal system to examine how the microbiota-gut-brain axis shapes the emergence of parental motivation.

**Results:**

Using zebra finches (*Taeniopygia guttata*), which exhibit sexually dimorphic nest-construction behaviors, we showed strong association between these preparatory parenting behaviors and gut microbiota composition. High-throughput sequencing to gut contents revealed female-specific convergent composition of gut microbiota when entering nesting status, with *Campylobacteraceae* rising as the predominant family. The gut microbiome of nesting birds showed enriched functions for energy metabolism and biosynthesis of essential amino acids and vitamins, reflecting elevated energy and nutritional demands, especially in females. We also observed sex-specific correlations between nesting actions and gut microbial diversity and *Lactobacillaceae* abundance. Notably, expression of social and gonad-related hormone genes in nesting-associated brain regions correlated with both microbial diversity and *Lactobacillaceae* abundance, suggesting integrated relationships between the gut microbiome, brain gene expression and nesting behavior.

**Conclusions:**

Our study integrates behavior experiments, microbiota profiles, and brain gene expression to investigate the role of gut microbiome in programming sex-specific nest construction behavior in birds. These findings suggest potential mechanisms through which the microbiota-gut-brain axis regulates parenting behaviors in avian systems, expanding our understanding of how gut microbiota influence complex behaviors across animal lineages.

**Supplementary Information:**

The online version contains supplementary material available at 10.1186/s42523-025-00486-w.

## Introduction

Animals live with diverse communities of symbiotic microorganisms. The microbial communities in intestines, which are shaped by host-specific factors (e.g., sex, physiological state and stress levels) and environmental conditions (e.g., diet and social interactions), may exert effects on local and distant organs [1, 2]. These gut microbiota influence not only the hosts’ physiological health but also their mental states and behaviors [3–5]. Recently, scientists found evidence supporting the microbiota-gut-brain axis, which integrates gut and central nervous activities through bidirectional neurohumoral communication mediated by gut microbiota [4, 6, 7]. Studies in rodents have demonstrated that gut microbiota affect behavior through neurological modifications. For instance, germ-free or antibiotic-treated mice exhibit reduced anxiety-like behavior but deficits in social behavior [8–10], and anxiety behavior can be transmitted between mice through gut microbiota transplantation [11]. In addition, administering probiotics, particularly *Lactobacillus* strains, has led to notable improvements in anxiety and social behavior in mice [7, 12, 13]. Recent investigations have uncovered that gut microbiota communicate with the brain via multiple routes, including neural, immunologic, endocrine, and metabolic pathways, thereby influencing neural function and behavior [4, 6, 14, 15]. These findings suggest that gut microbial signals play a crucial role in programming social behaviors in animals.

The evolution of host-microbe symbiosis has likely shaped how gut microbiota influence host physiology and behaviors [4, 6, 16]. This relationship appears mutually beneficial, as gut microbes can enhance their transmission by modulating host social behaviors. For example, in eusocial insects like honey bees and termites, increased social interactions through brood care and feeding behavior promote microbial transfer between colony members [17, 18]. From the perspective of hosts, this enriched microbial diversity may improve health and social interactions, including breeding behavior. However, the majority of evidence for the link between gut microbiota and the social brain has focused on rodent models, particularly mice, and highly social animals like primates and eusocial insects (e.g., [4, 6, 17, 18]). Limited knowledge exists regarding the microbiota-gut-brain axis in non-model vertebrates and its broader influences on various social interactions.

Among the social behaviors, parental care is particularly critical for offspring survival and thus under strong selection. It comprises an array of species-specific actions—from nursing pups in mammals to incubating eggs and brooding chicks in birds, along with nest construction or burrow creation—and is recognized as an innate or induced behavior [19–21]. Parenting behaviors typically emerge before offspring arrival, as individuals prepare for their future parental roles [20, 22, 23]. However, most studies on gut microbiota and social behaviors have predominantly focused on parenting behaviors that are enhanced by offspring presence or parent-offspring interactions (e.g., [24–26]). Nest-building behavior—an early parenting behavior that occurs prior to offspring-associated cues—presents a unique opportunity to investigate how the microbiota-gut-brain axis influences the initial emergence of parental motivation.

In birds, nest building often involves a cooperative effort between mates, or sometimes nests serve as courtship displays to attract potential mates [27, 28]. Once offspring are present, the nest becomes a focal point for parent-offspring interactions [24, 29]. These interactions underscore the significance of nesting behavior in promoting social contact among conspecifics [30]. The close relationship between nesting behavior and social interactions in birds provides an excellent system for investigating the avian microbiota-gut-brain axis, an emerging field with relatively few studies (e.g., [31, 32]). Such research promises to deepen our understanding of the interplay between microbial community ecology and animal behavior and illuminate the evolutionary processes involved. Our previous study supports this notion, indicating that parent zebra finches (*Taeniopygia guttata*) and society finches (*Lonchura striata domestica*) may transmit gut microbes to the chicks they raised via their feces within the nests [24]. Similar nest-mediated maternal transmission has recently been found in great tits (*Parus major*) and blue tits (*Cyanistes caeruleus*; [29]). These findings highlight how nesting behavior facilitates gut microbiota transmission within avian populations. Thus, we hypothesize that specific gut microbes may enhance nest-building behavior in birds since this would promote their transmission among adult and juvenile birds utilizing the nest.

In this study, we aim to examine the relationship between intestinal bacteria and nesting behavior using zebra finches as our avian model under controlled dietary and environmental conditions. The zebra finch forms monogamous pair bonds during breeding with male and female birds collaborating and taking on different nest-building tasks—males primarily collect nest materials while females mainly construct the nest at the chosen site with male assistance [28, 33]. The sex-specific division of nest-building tasks offers an excellent model for addressing how gut bacteria differently influence male and female actions. While Fang et al. [33] provided important neurotranscriptomic insights into nesting behavior in zebra finches, the potential role of gut microbiota in shaping these behavioral transitions has not been explored. Here, we address this knowledge gap by conducting a series of behavioral experiments on nesting and non-nesting finches, integrating gut microbiome profiling, functional pathway prediction, and brain gene expression data to identify candidate symbiotic microbes involved in avian nesting behavior. Specifically, we examine (1) whether transitions to nesting status and performance of nesting actions coincide with shifts in gut microbial community composition and dominant bacterial taxa; (2) whether observed microbial shifts correlate with altered functional potentials and changes in host brain gene expression; (3) whether these microbiota-behavior associations exhibit sex-specific patterns. Our findings will shed light on the connections between gut microbiota and early parenting behavior in birds, enhancing our understanding of the microbiota-gut-brain axis in non-model species.

## Methods

### Animals

Zebra finches used in this study were purchased from commercial bird stores in Taipei, Taiwan and acclimated for at least one month in the bird facility at Biodiversity Research Center, Academia Sinica before experimentation. The bird facility consisted of separate resting and breeding rooms maintained at 24 ± 4 °C and 50 ± 20% relative humidity, following conditions suggested by Mak et al. [34]. Prior to pairing and nesting behavior tests, birds were housed by sex in resting cages (100 × 100 × 100 cm) in the resting room under a 13/11 h light–dark cycle. The birds had access to finch seed mix consisted of white millet and canary seed (50% and 50% by volume), supplemented with niger seed (20 g per liter), and water *ad libitum*. The care and use of birds were approved by the Institutional Animal Care and Utilization Committee of Academia Sinica (Protocol ID: 17-05-1096).

### Nesting behavior experiment and sampling

To initiate the experiment, we transferred finches from the resting cages and randomly paired them (one male, one female) in breeding cages (45.5 × 37 × 38 cm) in the breeding room with a longer-day cycle (14/10 h light-dark photoperiod, lights on 5 AM), maintaining the same temperature and humidity as the resting room. Each cage contained an open nest box (15 × 15 × 3.5 cm). To ensure that all pairs had experiences of nest building, each pair received 10 g of coconut fibers as nest materials daily and we monitored nest material usage until they completed the process of nest building in the nest box. After this preparatory phase, we moved birds back to sex-separated resting cages for at least one week before formal experiments began.

**Fig. 1:**
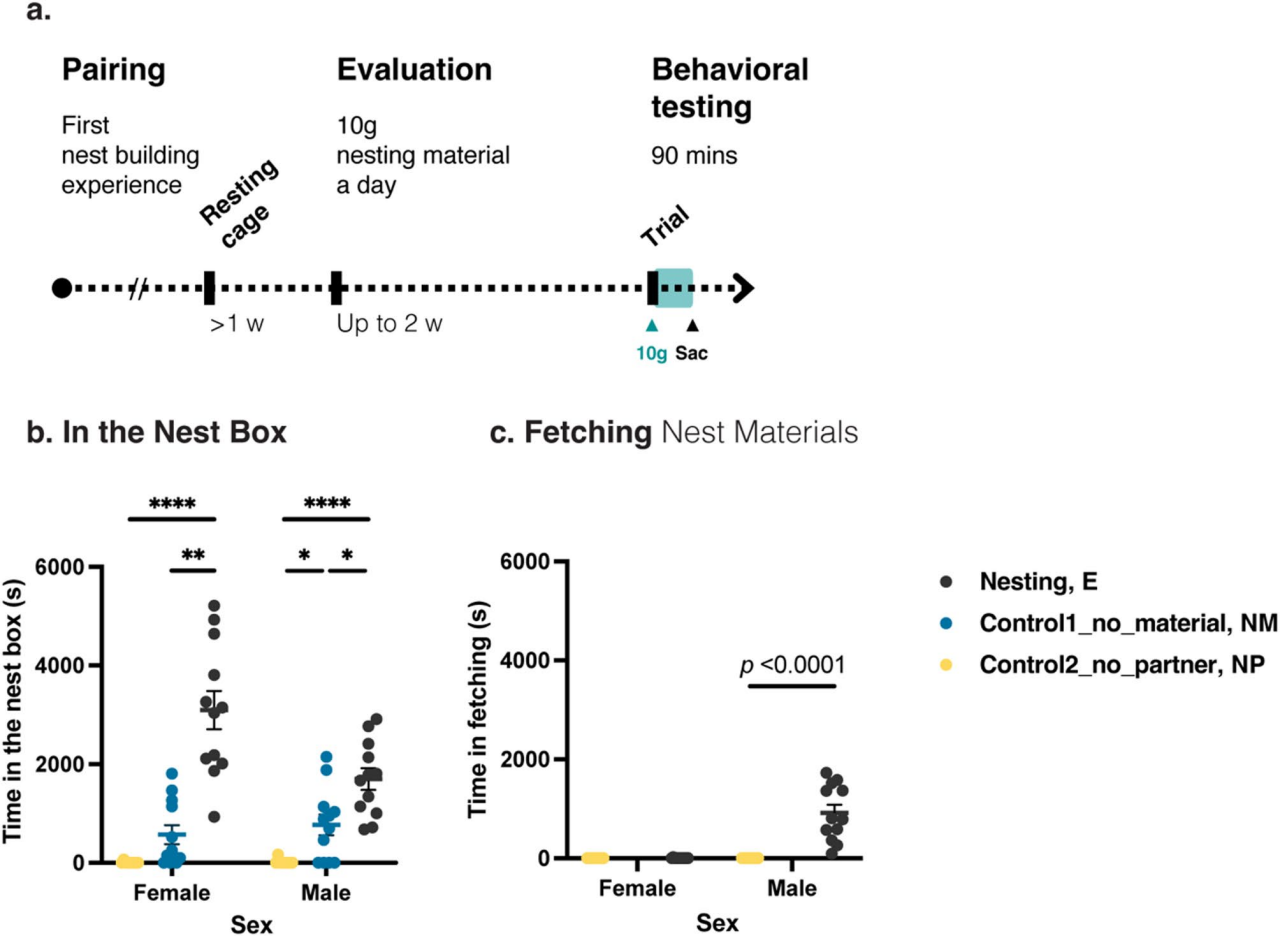
Nesting behavior experiment and nesting action analysis across different treatment groups. (**a**) Nesting behavior experiment design (**b**) comparison of time spent in nest box and (**c**) comparison of nest material fetching duration. The behavior experiment consists of three phases: preparatory pairing and nesting phase, nesting-ready status evaluation phase, and behavior testing phase (nesting behavior confirmation). Treatment groups are defined based on access to nesting cues: **E** = nesting group with paired partners and nest materials, **NM** = control group without nest materials (partners only), and **NP** = control group without partners (nest materials only). Plots show data points along with group means ± standard errors. Kruskal-Wallis tests indicate significant inter-group difference in time spent in the nest box (female: *p* < 0.0001; male: *p* < 0.0001) and material fetching (male: *p* < 0.0001). Pairwise differences in nest box staying time are evaluated with Dunn’s post-hoc tests (* *adj. p* < 0.05, ** *adj. p* < 0.01, **** *adj. p* < 0.0001)

To evaluate the association between gut microbiota and nesting behaviors, we analyzed samples from nesting behavior experiments described in Fang et al. [33], with most bird samples shared between both studies (*n* = 9−12 pairs each treatment group; Fig. 1a). For each experimental cohort, we randomly selected three pairs of birds that had previously been paired and successfully built nests (see above), and assigned them to different treatments: one nesting experimental pair (E) receiving nest materials, and two non-nesting control pairs (NM, without nest materials; NP, with the paired partners separated into two cages and each receiving nest materials). All cages of the three treatment groups contained a nest box, with E and NP groups receiving 10 g of nest materials daily. At 9 AM each day, we examined nesting (E) pairs to determine their “nesting-ready status”. Progression to the subsequent experimental phase occurred only when both the following two criteria were fulfilled: (1) observed deposition of nest materials inside the nest box, and (2) either the complete consumption of the newly supplied 10 g of materials or the consistent utilization of a minimum of 30% (≥ 3 g) per day for three consecutive days. Unused materials remained from the previous day were removed and weighed each morning to quantify daily material usage. Upon reaching these criteria, we performed a “nesting behavior confirmation” test that same morning. The “nesting behavior confirmation” test began at 10 AM, with E and NP groups receiving 10 g of nest materials and all groups video-recorded using IP network cameras (AW-720CIP, Jinwei Electronic Co., Ltd., Taipei, Taiwan) for 90 min (between 10:00−11:30 AM). We observed whether E pairs exhibited sex-specific nesting actions—male fetched and deposited materials into the nest box and the female stayed in the nest box (indicating nest construction; [35–37]). If these nesting actions did not occur within the 90 min of behavior confirmation test, the test was repeated the following morning.

If the whole nesting behavior test could not be completed within a two-week period, the experiment was terminated and all pairs were moved back to the resting cages for at least one week before a next round of experiments. Among 21 attempts, seven failed to complete in the two-week testing period. Once the E group reached the criteria, all birds from the three treatment groups were sacrificed 30–60 min after the confirmation test (12:00–12:30 PM). Gut contents of finches were collected and frozen immediately in dry ice and stored at −80 °C until DNA extraction. Their brain tissues were collected for transcriptomic analysis (detailed in Fang et al. [33], with relevant transcriptomic data incorporated into the present study (see the last section of Methods).

### Nesting behavior quantification

We analyzed the 90-minute behavioral recordings from the morning of gut content sampling (i.e., E pairs entering nesting status) to quantify nesting actions across all three treatment groups (E, NM and NP). We focused on two key actions—fetching nest materials to the nest box and staying in the nest box (as a proxy of nest construction; [35–37])—and measured the duration of each action. We compared the time spent in the nest box across three treatment groups using Kruskal-Wallis tests (for three-group comparisons) followed by Dunn’s post-hoc tests (for pairwise comparisons) and in fetching nest materials using Kruskal-Wallis tests (for E-NP comparisons) implemented in the R package *FSA* [38]. Additionally, to evaluate variations in nesting motivation among E pairs, we used the latency to initiate nesting—defined as the number of days the E pairs took to meet the nesting behavior confirmation criteria—as a proxy for their motivation to enter nesting status.

### DNA extraction, 16S amplicon library preparation and sequencing

We homogenized the collected gut contents in 1 mL of lysis buffer (inhibitEX Buffer, QIAamp Fast DNA Stool Mini Kit, Cat. No. 51604, QIAGEN, Germany) in Precellys Lysing Kit CK14 tubes (Bertin Technologies, France) using FastPrep-24 Homogenizer (MP Biomedicals). We extracted bacterial DNA from homogenized samples using the QIAamp DNA Stool Mini Kit and quantified it using a Qubit 2.0 Fluorometer (Invitrogen, Life Technologies, USA). We amplified the first two hypervariable regions (V1-V2) of the small subunit ribosomal RNA (16S rRNA) gene from the extracted DNA using universal eubacterial primers. The forward primer 27F (5′-AGAGTTTGATCMTGGCTCAG-3′) and reverse primer 355R (5′-GCTGCCTCCCGTAGGAGT-3′) were fused with Illumina overhang adapters and specific 10-nt barcodes to allow multiple samples to be analyzed in parallel on a single flow cell. The purified amplicons were further processed according to the Illumina standard protocol, and paired-end 2 × 300 bp sequencing was conducted on the MiSeq platform (Illumina, USA) with the reagent kit v3 at the NGS High Throughput Genomics Core Facility, Academia Sinica. A full list of sample metadata is provided in Table S1.

### Sequence data processing

We trimmed V1/V2 primer sequences from raw amplicon Illumina sequencing data using the *cutadapt* plugin in QIIME 2 version 2022.2 [39]. Based on quality assessments, we truncated forward and reverse sequencing reads at 200 and 160 bases, respectively, before merging and denoising into amplicon sequence variants (ASVs) using the Divisive Amplicon Denoising Algorithm 2 (DADA2) [40]. We excluded ASVs present in fewer than two samples and with less than 137 counts (0.1% of the mean sequencing depth), and assigned taxonomy using the Silva 138 database (SSU Ref NR 99; [41]). We also removed ASVs classified as chloroplasts and mitochondria from the dataset using the QIIME2 taxa filter-table command, as they likely represented ingested plant materials.

### Gut microbial community diversity

We randomly picked 15,000 reads from each sample to estimate the gut microbial community diversity. To estimate alpha diversity, representing within-host (within a bird’s gut content) diversity, we computed two metrics—Shannon index and Faith’s phylogenetic diversity (Faith’s PD)—using QIIME 2. We compared the alpha diversity metrics across zebra finch treatment groups using Kruskal-Wallis tests (for three-group comparisons) followed by Dunn’s post-hoc tests (for pairwise comparisons) implemented in the R package *FSA*. We also estimated beta diversity to assess variation in microbial community structure among hosts using two metrics—Bray-Curtis dissimilarity and weighted UniFrac distance [42]. Microbial community structure was visualized using principal coordinates analysis (PCoA) for both beta diversity measures. To assess differences in beta diversity across the treatment groups, we used two non-parametric multivariate approaches—analysis of similarity (ANOSIM) [43] and permutational multivariate analysis of variance (PERMANOVA/Adonis) [44].

### Differential abundance of gut microbial taxa among treatment groups

We conducted Linear Discriminant Analysis Effect Size (LEfSe) analysis [45] to assess the enrichment of gut microbial taxa in any of the three treatment groups. In further details, we identified individual taxa that showed differential abundance in one of the three treatment groups against each of the other two based on sequential applications of Kruskal-Wallis tests (for three-group comparisons) followed by Wilcoxon rank-sum tests (for pairwise comparisons). Linear Discriminant Analysis (LDA) was then applied to the identified taxa to estimate their effect sizes for inter-group variation based on the first discriminant axis. We adopted a test significance level at *p*-value < 0.05 and reported enriched taxa for specific groups that gained absolute values of the log10 effect sizes (the LDA scores) >2.0.

### Predictive metagenome functions of gut microbiota in treatment groups

We used a bioinformatic tool, Phylogenetic Investigation of Communities by Reconstruction of Unobserved States (PICRUSt2) analysis [46], to predict the functional potentials of gut microbiome. PICRUSt2 placed ASVs into a reference phylogeny to predict the abundance of their gene families based on the Enzyme Commission (EC) number database, and mapped the EC gene families to metabolic pathways from the MetaCyc database [47]. Based on the mapped pathways, we performed the LEfSe analysis to determine enriched pathways in finch groups under specific treatments with a significance threshold of *p*-value < 0.05 and filtered with an LDA score of 2. The enriched metabolic pathways were examined at MetaCyc Superclass1 and Superclass2 categorical levels.

### Correlations between gut microbial features and nesting actions

Given that nest-building behavior in zebra finches involves a series of movements with sexual differences, it is possible to examine associations between gut bacteria and individual actions—fetching nest materials and staying in the nest box. Additionally, the design of our nesting behavioral experiment allowed us to assess whether finches differ in their latency to initiate nest construction—a measure of baseline motivation to nesting. We examined relationships between these nesting actions (material fetching, nest box occupancy, and construction initiation latency) and gut microbial features (i.e., alpha diversity metrics and the relative abundance of bacterial taxa) among finches from the E group using Spearman’s rank correlation in GraphPad Prism version 10.3.0 for macOS.

### Correlations between finch gut microbial features and brain gene expression

To investigate gut microbiota-brain interactions during nest construction in zebra finches, we analyzed correlations between gut microbial features and brain gene expression using transcriptome data from Fang et al. [33]. The brain transcriptome came from four brain regions that are associated with nest-construction behavior [33]: the anterior motor pathway (AMP), the social behavior network (SBN), and the dopaminergic reward system (dopaminergic neuron population, DNP)—as their neuronal activities are related to nest-building behavior in zebra finches [35, 48]—as well as the pons and medulla (PM)—which convey tactile-related sensorimotor signals from and to the beak [49] and gut-induced signals from the vagus nerve [50]. Analysis included individuals that contained both gut microbiome and brain transcriptome data (*n* = 5 pairs for each treatment group; Table S2).

We selected 20 genes of interest (GOI) for the analysis based on their established roles in reproductive and social behaviors. As Fang et al. [33] showed that the expression of social hormone genes was related to the nesting behavior of zebra finches, we chose 17 GOI associated with social hormones—oxytocin (*oxt*), vasopressin (*avp*), arginine vasopressin-induced protein 1 (*avpi1*), vasoactive intestinal peptide (*vip*), dopamine (*th*), tachykinin precursor 1 (*tac1*), and their receptors—from their brain transcriptome dataset (see Table S3 for details). In addition, considering that sex hormones and gonadal maturation are related to bird nest building [51,53], we chose another three GOI—gonadotropin-releasing hormone (*gnrh1*) and its receptor (*gnrhr*), as well as protein tyrosine phosphatase non-receptor type 5 (*ptpn5*). The three GOI are expressed in the brain and function together to influence sex hormone secretion and gonad maturation [54] as well as fish nesting behavior [55]. Notably, 12 of our 20 GOI were previously identified as differentially expressed genes between the nesting (E) and non-nesting (NP/NM) zebra finch groups [33]; Table S3), providing additional evidence for their functional relevance to nest construction behavior. Subsequently, we normalized read count data based on the median-of-ratios method [56] for conducting Spearman’s rank correlation analysis using the R package *DESeq2* [57]. We examined Spearman’s correlation between expression of the 20 GOI in the four brain regions and gut microbial features (i.e., alpha diversity metrics and the relative abundance of taxa) using GraphPad Prism version 10.3.0 for macOS. We performed the analysis on samples from all three treatment groups combined and the E group, separately. To highlight correlations with strong statistical confidence, we applied the Benjamini–Hochberg correction to *p*-values to account for multiple comparisons based on the numbers of GOI using the R command (p.adjust).

## Results

### Divergent nesting actions between the experimental and control groups

We compared nesting behaviors across the normal pairing experimental group (E, *n* = 12 pairs) and the two control groups, including pairs without access to nest materials (NM, *n* = 12 pairs) and pairs separated from partners (NP, *n* = 9 pairs; Table S2). We recorded their nesting actions after the E-group finches entered the nesting status (Fig. 1a). Female finches in the E group spent significantly longer time in the nest box than those in each control group (E-NM *adj. p* < 0.0001, E-NP *adj. p* = 0.0023, Dunn’s post-hoc tests; Fig. 1b). Male finches showed a similar trend, with E-group males staying in the nest box longer than NP- and NM-group males (E-NP *adj. p* < 0.0001, E-NM *adj*. *p* = 0.0389; Fig. 1b). Males exclusively fetched nest materials to the nest box, and the E-group males dedicated significantly more time than the NP-group ones (E-NP *p* < 0.0001, Kruskal-Wallis test; Fig. 1c). Overall, both females and males in the E group exhibited higher levels of nesting actions than those in the control groups.

### Gut microbiome diversity differences between nesting and non-nesting birds

To examine the links between gut microbiota and nesting status, we collected the gut contents of zebra finches from the nesting group (E, *n* = 9 pairs) and the two non-nesting control groups (NM, *n* = 9 pairs; NP, *n* = 6 pairs). The application of 16S rRNA amplicon sequencing on the 48 zebra finches generated a total of 5,791,186 reads after quality filtering (23,856 to 255,889 reads per sample), which were used to construct 142 ASVs.

We first compared alpha (within-host ASV) diversity levels across the three treatment groups using two metrics, Shannon index and Faith’s PD. Combining data from both sexes showed no significant differences among the three treatment groups regardless of the diversity metrics used (Shannon index: H = 2.1610, *p* = 0.3394, Faith’s PD: H = 5.7647, *p* = 0.0560, Kruskal-Wallis tests; Fig. S1). Nevertheless, female-specific analysis revealed that Shannon diversity was significantly reduced in the E group compared to the NM group (H = 6.2056, *p* = 0.0449; E-NM *adj. p* = 0.0449, Dunn’s post-hoc test), but did not differ significantly between the E and NP groups (E-NP *adj*. *p* = 0.2350; Fig. S1a). The Shannon index-based comparisons showed no significant differences among treatment groups for male birds (H = 0.4356, *p* = 0.8043, Kruskal-Wallis tests; Fig. S1a), nor did Faith’s PD-based comparisons among either female or male groups (female: H = 3.9389, *p* = 0.1395; male: H = 3.4056, *p* = 0.1822; Fig. S1b).

**Fig. 2:**
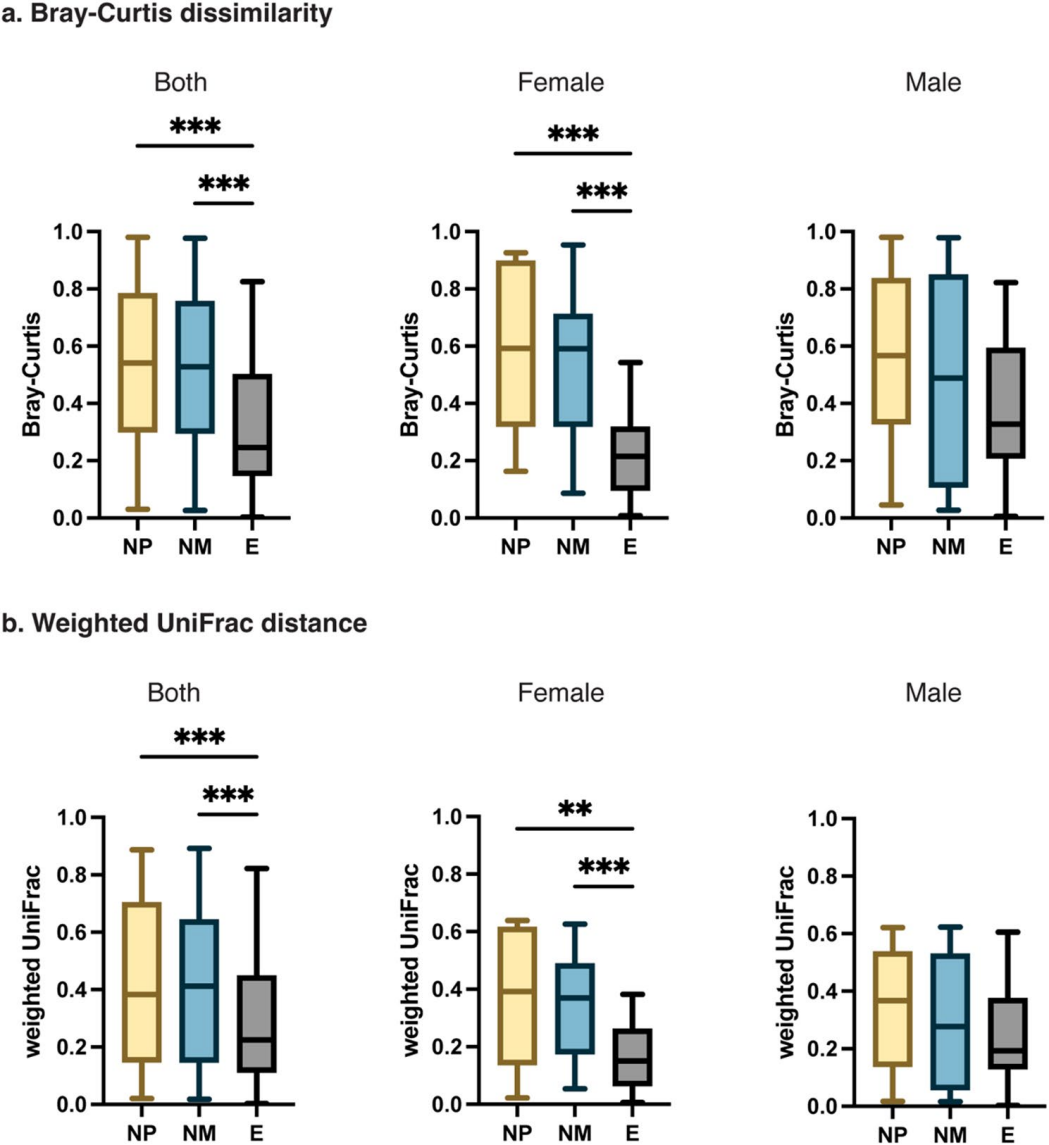
Beta diversity of gut microbial communities across different treatment groups. Beta diversity among samples is estimated based on (**a**) the Bray-Curtis dissimilarity and (**b**) the weighted UniFrac measure. Levels of beta diversity are compared among three treatment groups using non-parametric Kruskal-Wallis tests followed by Dunn’s post-hoc tests (** *adj*. *p* < 0.01, *** *adj*. *p* < 0.001). **E** = birds with paired partners and nest materials (nesting group); **NM** = birds with partners only (control: no materials); **NP** = birds with nest materials only (control: no partners)

We then used the Bray-Curtis and the weighted UniFrac measures to assess ASV compositional variation among individual samples (i.e., beta diversity). We detected significant compositional differences among the three treatment groups based on both measures (Bray-Curtis: pseudo-F = 3.892, *p* = 0.016, weighted UniFrac: pseudo-F = 3.3669, *p* = 0.030, PERMANOVA; Table S4). Likewise, we detected significantly greater variation among treatment groups than within groups (Bray-Curtis: *R* = 0.0954, *p* = 0.021, weighted UniFrac: *R* = 0.0862, *p* = 0.040, ANOSIM). To identify which group differentiated from the others, we visualized inter-sample compositional variation using principal coordinate analysis (PCoA). We found a major distinction between the nesting group and the two control groups along PCo1 (Fig. S2). Noticeably, the E-group birds showed significantly less variation in gut microbial beta diversity than the NM- and NP-group birds (Bray-Curtis: H = 49.95, *p* < 0.001, weighted UniFrac: H = 23.87, *p* < 0.001, Kruskal-Wallis tests; Bray-Curtis: E-NM *adj. p* < 0.001, E-NP *adj. p* < 0.001, weighted UniFrac: E-NM *adj. p* < 0.001, E-NP *adj. p* < 0.001, Dunn’s post-hoc tests; Fig. 2). When finches of different sexes were analyzed separately, the beta diversity patterns described above held for the female birds (Bray-Curtis: H = 30.540, *p* < 0.001, weighted UniFrac: H = 17.890, *p* < 0.001, Kruskal-Wallis tests; Bray-Curtis: E-NM *adj. p* < 0.001, E-NP *adj. p* < 0.001, weighted UniFrac: E-NM *adj. p* < 0.001, E-NP *adj. p* = 0.005, Dunn’s post-hoc tests) but not for the male ones (Bray-Curtis: H = 3.712, *p* = 0.16, weighted UniFrac: H = 1.447, *p* = 0.49, Kruskal-Wallis tests; Fig. 2).

In sum, zebra finches showed distinct gut microbiota compositions under different nesting conditions, with nesting females showing reduced alpha and beta diversity compared to non-nesting females.

### Dominant gut microbial taxa switched with the nesting status of finches

To identify whether specific microbes were enriched in different nesting conditions, we first analyzed the relative abundance of gut microbial lineages in nesting (E, *n* = 9 pairs) and non-nesting finches (NM, *n* = 9 pairs; NP, *n* = 6 pairs). For individual finches, the relative abundance of the phylum Firmicutes was lowest in the E group (group mean ± standard error = 15.5 ± 4.3%), followed by the NM (36.3 ± 8.4%) and then NP groups (50.9 ± 11.4%), ranging from 0.2% to 97.1%. The phylum Campilobacterota (reclassified from the proteobacterial class Epsilonproteobacteria) showed the highest relative abundance in the E group (77.2 ± 5.7%), followed by the NM (58.9 ± 36.3%) and then NP group (46.4 ± 38.5%), ranging from 1.8% to 99.8%. The phylum Proteobacteria was relatively less abundant across groups (E: 6.9 ± 4.2%, NM: 4.4 ± 2.5% and NP: 1.5 ± 0.6%), ranging from 0% to 71.3% (see all metadata in Table S1).

**Fig. 3:**
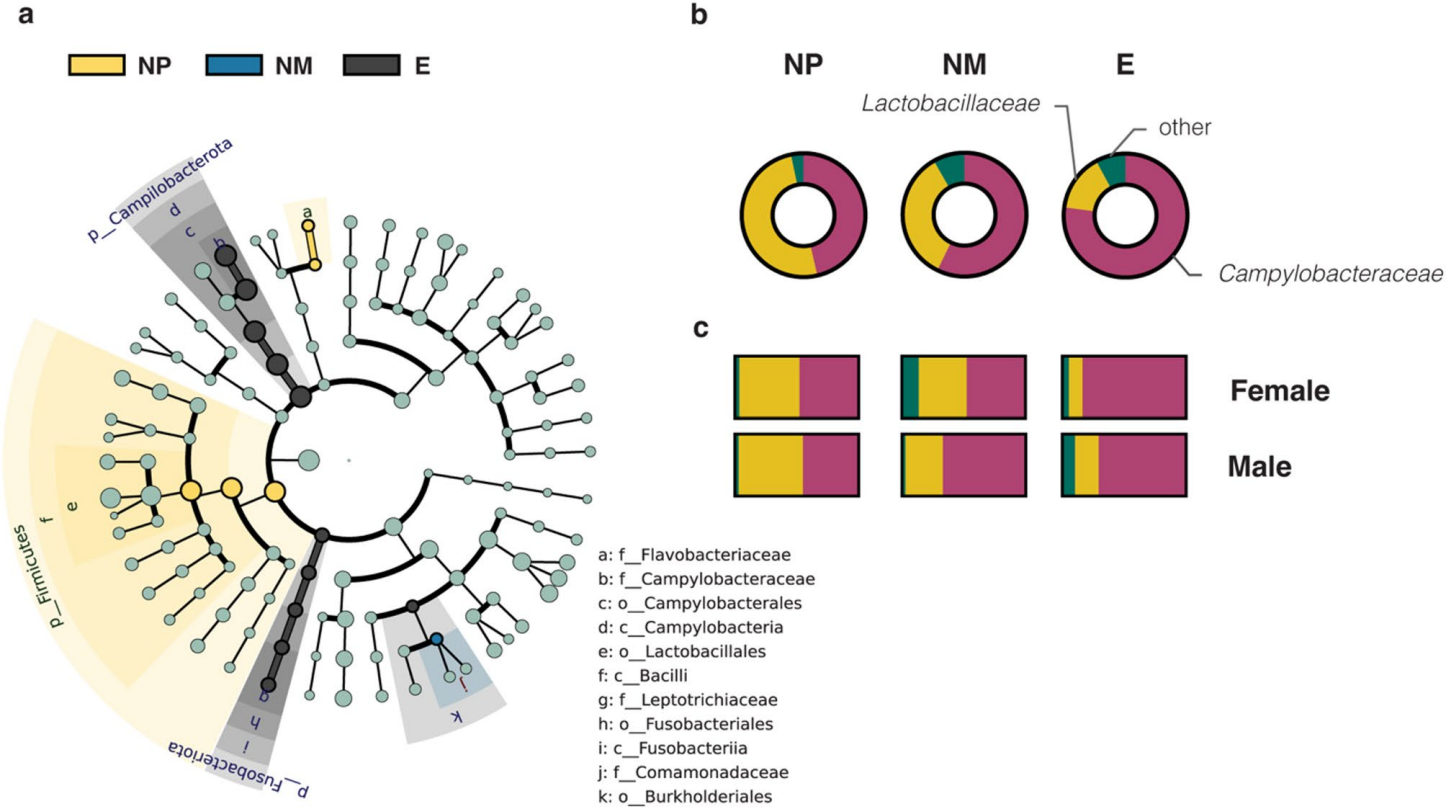
Dominant gut bacterial lineages in different treatment groups. (**a**) Enrichment of gut bacterial taxa in birds under specific treatments is evaluated by the linear discriminant analysis effect size (LEfSe) analysis along bacterial taxonomic ranks from phylum to genus (from the tree center to edge). Significance is determined with criteria of *p* < 0.05 and LDA score > 2.0. Relative abundance of families *Campylobacteraceae* and *Lactobacillaceae* in the gut bacteria when finches of the two sexes are examined together (**b**) or separately (**c**). **E** = birds with paired partners and nest materials (nesting group); **NM** = birds with partners only (control: no materials); **NP** = birds with nest materials only (control: no partners)

Next, we performed the LEfSe analysis to identify 17 bacterial taxa, with taxonomic ranks from phylum to genus, showing significant enrichment (*p* < 0.05, LDA score > 2) in specific treatment groups (Fig. 3a). Specifically, three taxa, *Campylobacteraceae* (family), *Leptotrichiaceae* (family), and *Burkholderiales* (order), and eight higher taxonomic ranks containing these taxa were significantly enriched in the E-group birds. Another two taxa, *Flavobacteriaceae* (family) and *Lactobacillales* (order), together with three higher taxonomic ranks containing them were significantly enriched in the NP-group birds. Finally, the bacterial taxon *Comamonadaceae* (family) was significantly enriched in the NM-group birds.

When we focused on the family level, two predominant bacterial lineages appeared—*Campylobacteraceae* and *Lactobacillaceae* (Fig. 3b). *Campylobacteraceae* tended to be more abundant in the nesting birds than in the non-nesting control groups (H = 6.5313, *p* = 0.0382, Kruskal-Wallis test; E-NM *adj. p* = 0.1106, E-NP *adj. p* = 0.0535, Dunn’s post-hoc tests), and *Lactobacillaceae* showed a non-significant opposite trend (H = 5.1198, *p* = 0.0773; Figs. 3b and S3). Notably, we found that the enrichment of *Campylobacteraceae* in nesting compared to non-nesting birds was more prominent in females (H = 7.7600, *p* = 0.0207; E-NM *adj. p* = 0.0280, E-NP *adj. p* = 0.0793) than males (H = 1.7156, *p* = 0.4241; E-NM *adj. p* = 0.7389, E-NP *adj. p* = 0.5995; Figs. 3c and S3). Overall, the two dominant bacterial families showed opposite trends of relative abundance change across nesting conditions with stronger patterns found in female birds.

### The functional potentials of gut microbiota differed by the nesting status of finches

**Fig. 4:**
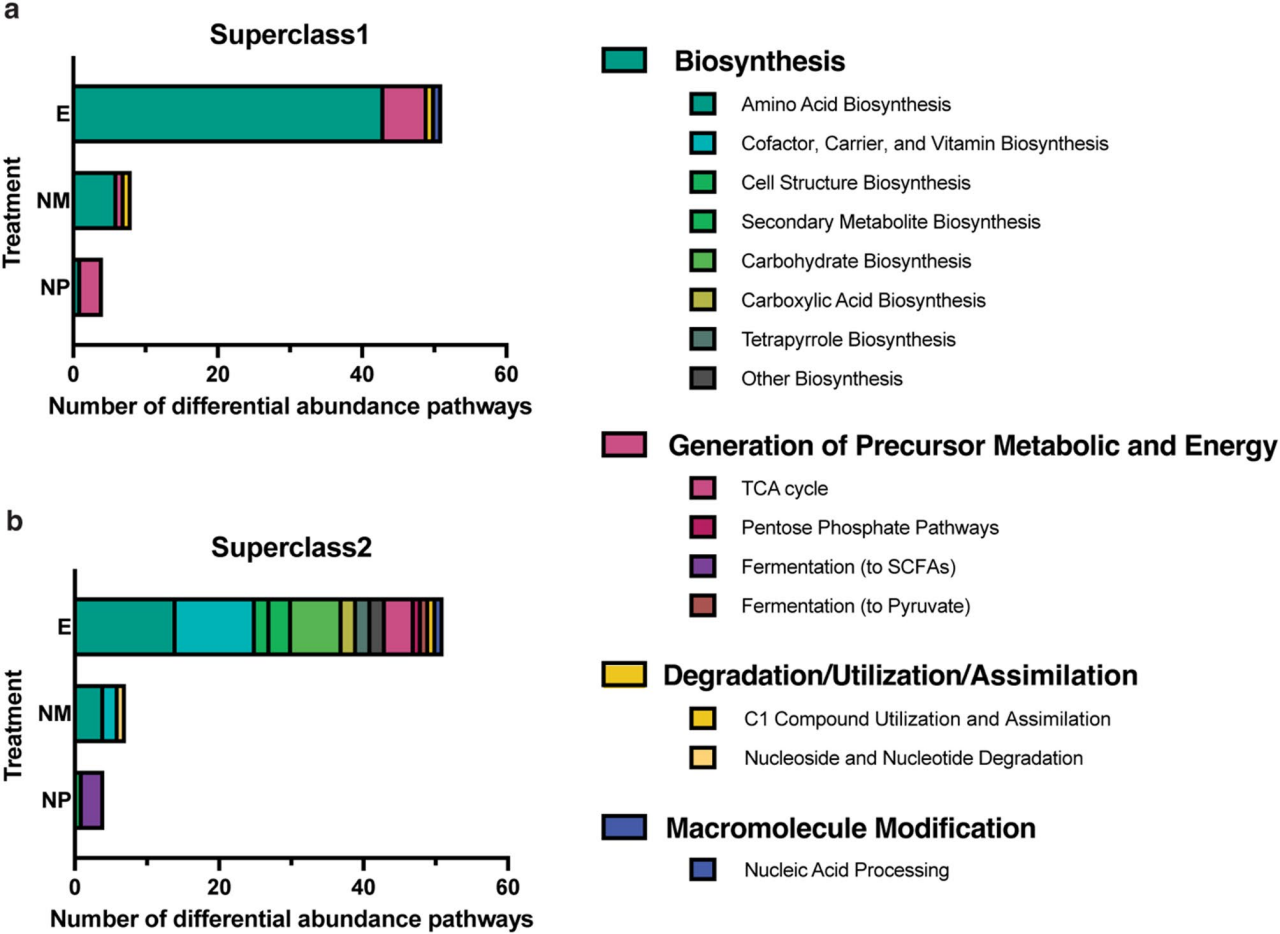
Enriched functional pathways of gut microbiota across treatment groups. PICRUSt2 pathways are categorized at MetaCyc (**a**) Superclass 1 and (**b**) Superclass 2 levels with enrichment determined by the linear discriminant analysis effect size (LEfSe) analysis. Treatment groups were defined based on access to nesting cues: **E** = nesting group with paired partners and nest materials, **NM** = control group without nest materials (partners only), and **NP** = control group without partners (nest materials only). Significance is determined with criteria of *p* < 0.05 and LDA score > 2.0

To estimate potential differences in microbiota-driven metabolites across nesting conditions, we used PICRUSt2 to identify 278 enriched MetaCyc functional pathways of the gut microbiota, of which 63 were discriminative among the three treatment groups (*p* < 0.05, LDA score > 2; Fig. S4 and Table S5). These 63 pathways belonged to four MetaCyc primary classes (Superclass 1) and 15 subclasses under these primary classes (Superclass 2; Fig. 4). Among Superclass 1 categories, ‘Biosynthesis’ and ‘Generation of Precursor Metabolites and Energy’ showed the largest variation among treatment groups (Fig. 4a). In the ‘Biosynthesis’ primary class, 43 pathways were significantly enriched in the E group (Fig. S4), including 14 ‘Amino Acid Biosynthesis’ pathways, 11 ‘Cofactor, Carrier, and Vitamin Biosynthesis’ pathways, seven ‘Carbohydrate Biosynthesis’ pathways, three ‘Secondary Metabolite Biosynthesis’ pathways and two in each of the ‘Cell Structure Biosynthesis’, ‘Carboxylic Acid Biosynthesis’, ‘Tetrapyrrole Biosynthesis’ and ‘Other Biosynthesis’ subclasses. Four ‘Biosynthesis’ pathways were significantly enriched in the NM group, including three in the ‘Amino Acid Biosynthesis’ and one in the ‘Cofactor, Carrier, and Vitamin Biosynthesis’ subclasses. The NP group showed enrichment in one ‘Cell Structure Biosynthesis’ pathway (Fig. 4, Table S5). In the ‘Generation of Precursor Metabolic and Energy’ primary class, six pathways were significantly enriched in the E group (Fig. S4, Table S5), including four ‘TCA cycle’, one ‘Pentose Phosphate Pathways’ and one ‘Fermentation to Pyruvate’ pathways. Another three ‘Generation of Precursor Metabolic and Energy’ pathways associated with ‘Fermentation to Short-Chain Fatty Acids’ were significantly enriched in the NP group (Fig. 4, Table S5).

We revealed that the gut microbiome of finches transitioning to nesting status exhibited a metabolic signature profoundly distinct from their non-nesting counterparts, despite identical diets and cage environmental conditions. The bacteria of E-group finches showed enhanced potential for several pathways, particularly those related to the TCA cycle (e.g., TCA cycle I: LDA = 3.2159, *p* = 0.0305; Fig. S4) and the biosynthesis of essential amino acids, including aromatic (e.g., *L*-tryptophan biosynthesis: LDA = 3.2560, *p* = 0.0243; super pathway of aromatic amino acid biosynthesis: LDA = 3.2409, *p* = 0.0278) and branched-chain amino acids (e.g., *L*-isoleucine biosynthesis II: LDA = 3.2640, *p* = 0.0283), and the biosynthesis of vitamins (e.g., Flavin biosynthesis I: LDA = 3.1661, *p* = 0.0141; Phosphopantothenate biosynthesis I: LDA = 3.0672, *p* = 0.0432). In stark contrast, the microbial communities in the non-nesting controls were primarily characterized by metabolic pathways involved in short-chain fatty acid metabolism (e.g., homolactic fermentation: LDA = 3.4695, *p* = 0.0286) in the NP-group birds or the biosynthesis of arginine and ornithine (e.g., *L*-arginine biosynthesis I via *L*-ornithine: LDA = 3.4695, *p* = 0.0286; *L*-ornithine biosynthesis: LDA = 2.5908, *p* = 0.0204) in the NM-group birds.

Sex-specific functional analysis revealed distinct patterns. Female birds showed similar results as birds of both sexes combined, except that no enriched pathways were found in the NM group, while males showed few enriched pathways with only two in the NP group (Fig. S4). Overall, the gut microbiome of nesting zebra finches exhibited functional enrichment in energy generation and the biosynthesis of essential amino acids and vitamins, especially in female birds.

### Gut microbial diversity and taxa abundance correlated with finch nesting actions

**Fig. 5:**
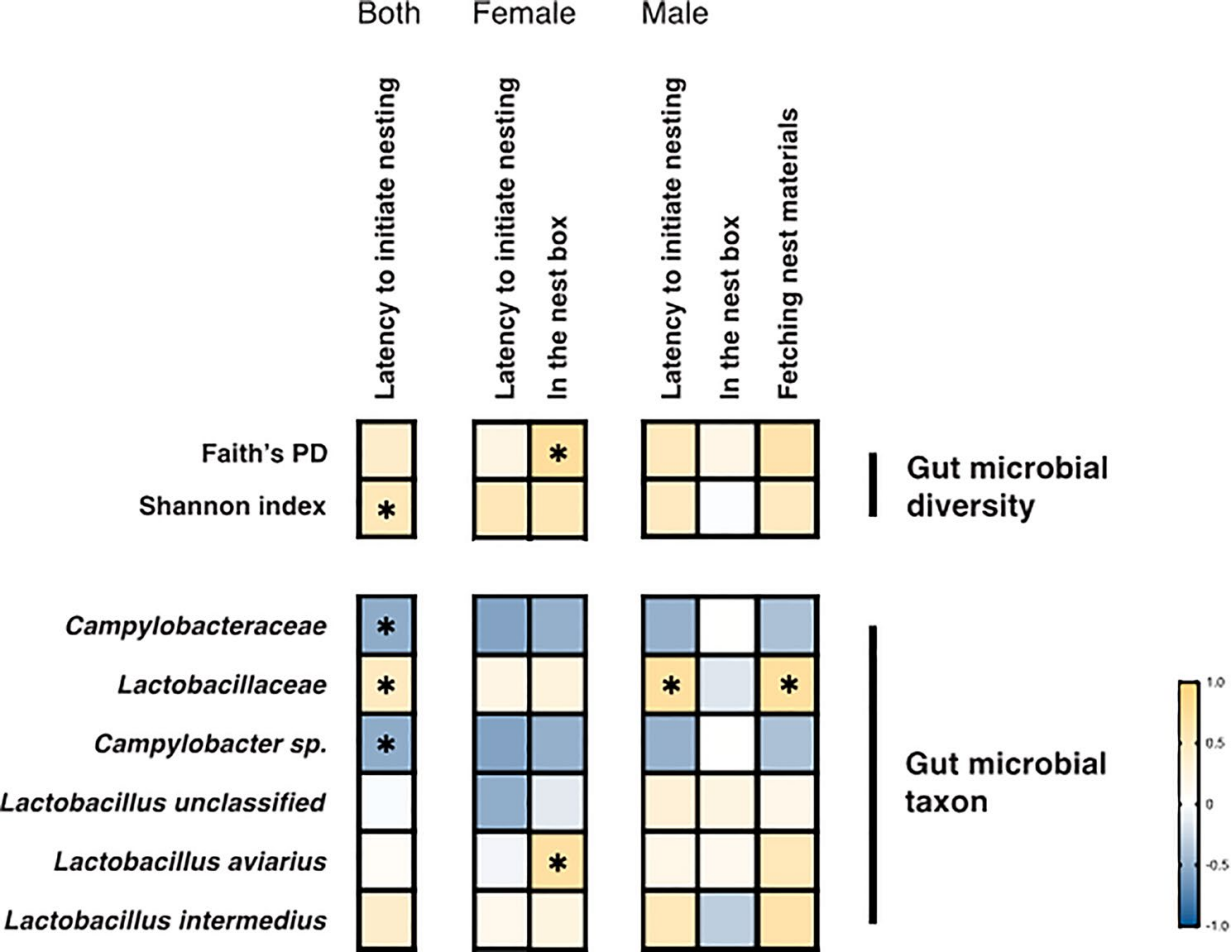
Correlations between gut microbial features and nesting actions among nesting zebra finches. Heat maps show Spearman’s rank correlation coefficient (ρ) between nesting actions and gut microbial alpha diversity as well as taxon abundance, analyzed separately for (**a**) all nesting individuals combined, (**b**) nesting females and (**c**) nesting males. Color intensity represents correlation strength, with yellow indicating positive correlations and blue indicating negative correlations (* *p* < 0.05)

We further examined correlations between gut microbial features (alpha diversity and taxon abundance) and specific nesting actions in the E group. Our analysis focused on three key actions: latency to initiate nesting, staying in the nest box, and fetching nest materials. Latency to initiate nesting exhibited significant positive correlations with gut microbial diversity (Shannon index: ρ = 0.548, *p* = 0.0186) and the abundance of the bacterial family *Lactobacillaceae* (ρ = 0.496, *p* = 0.0365; Figs. 5 and S5a, Table S6). In contrast, nesting latency was negatively correlated with the abundance of the *Campylobacteraceae* family (ρ = −0.525, *p* = 0.0253) and *Campylobacter sp.* (an unnamed species, ρ = −0.525, *p* = 0.0253; Figs. 5 and S5b, Table S6).

Sex-specific analyses revealed distinct patterns. In nesting females, the duration of staying in the nest box positively correlated with both gut microbial diversity (Faith’s PD: ρ = 0.750, *p* = 0.0255) and *Lactobacillus aviarius* abundance (ρ = 0.712, *p* = 0.0397; Fig. 5). Males exhibited significant positive correlations between *Lactobacillaceae* abundance and two nesting actions—fetching nest materials (ρ = 0.733, *p* = 0.031) and nesting latency (ρ = 0.731, *p* = 0.0305; Figs. 5 and S5c). However, the correlation between *Campylobacteraceae* abundance and fetching behavior was not statistically significant (ρ = −0.400, *p* = 0.2912; Fig. S5d).

### Gut microbial composition correlated with brain gene expression of social and gonadotropin-releasing hormones

To elucidate the potential mechanisms underlying microbial-mediated changes in nesting behavior, we examined the correlation between gut microbial features and the expression levels of 20 GOI across four brain regions (AMP, SBN, DNP, and PM). Among all birds from nesting and two non-nesting groups (*n* = 5 pairs for each group), we found significant negative correlations between the expression levels of *gnrhr* gene and Faith’s PD in AMP (ρ = −0.558, *adj. p* = 0.0256) and between *gnrh1* gene and the Shannon index in PM (ρ = −0.598, *adj. p* = 0.0095; Table S7). Moreover, the expression levels of two GOI—*vipr1* (ρ = 0.511, *adj. p* = 0.0371) and *ptpn5* (ρ = 0.519, *adj. p* = 0.0371)—significantly positively correlated with the Shannon diversity index in AMP. In sex-specific analysis, females showed several strong positive correlations, including those between *ptpn5* expression and Faith’s PD in AMP (ρ = 0.754, *adj. p* = 0.0330), between *avpi1* expression and the *Lactobacillaceae* family (ρ = 0.743, *adj. p* = 0.0434) as well as *Lactobacillus intermedius* abundance in SBN (ρ = 0.736, *adj. p* = 0.0500), and between *drd3* expression and *L. aviarius* abundance in SBN (ρ = 0.784, *adj. p* = 0.0198). In contrast, males only exhibited a significant positive correlation between *vip* and *L. aviarius* abundance in PM (ρ = 0.694, *adj. p* = 0.0440). Within the E-group birds, the *gnrhr* gene expression positively correlated with Faith’s PD in SBN (ρ = 0.877, *adj. p* = 0.0361). We found no significant correlations in the E-group males or females after multiple corrections. Together with the earlier findings, we showed that gut microbiota correlated with expression of social and gonad-related genes in brain regions that were associated with nesting behaviors in zebra finches.

## Discussion

The microbiome-gut-brain axis plays a crucial role in shaping social behaviors [4], yet its influence on parenting behavior remains largely unexplored. To address this gap, we examined how gut microbiota composition interacts with nesting behavior in zebra finches using a controlled experimental design that manipulated access to mates and nest materials. Our analysis revealed sex-specific differences in gut microbial communities and their associated functional pathways between nesting and non-nesting birds, with particularly distinct patterns in females. In addition, gut microbiota diversity and bacterial taxa abundance correlated with specific nesting actions differently than with overall nesting status, suggesting temporal dynamics in the microbiome-behavior relationship. By examining the interplay among nest-building behavior, gut microbiota profiles, functional pathways, and brain gene expression, we offer the first empirical evidence of the reciprocal interactions between the gut microbiome and bird nest construction. This study advances our understanding of the microbiota-gut-brain axis in the context of reproductive behavior.

### Convergent gut microbial composition upon zebra finches entering nesting status

Gut microbial diversity may modulate animal behavior through its interactions with host physiology [58–60]. Our comparison of gut microbiota between nesting and non-nesting finches revealed distinct patterns associated with nesting status. While the alpha diversity of gut microbiome remained largely similar between nesting and non-nesting birds, nesting female birds showed significantly reduced Shannon index compared to non-nesting females without access to nest materials. Notably, nesting birds displayed significantly reduced inter-group beta diversity in gut microbiota compared to both non-nesting control groups, indicating a convergence of gut microbial communities among individuals engaged in nesting behavior.

These findings are consistent with studies showing decreased gut microbial diversity during parental care—a continuous decline in alpha diversity in zebra finches [26] and lower alpha and beta diversity in nesting canaries (*Serinus canaria*) compared to resting ones [61]. Our results expand this narrative and suggest that early parenting behavior such as nest construction also reduces gut microbiota diversity. Given that higher microbial diversity often correlated with improved host health [59], the observed reduction during nesting may reflect physiological cost of parenting behavior. The resting period before reproduction may serve as a recovery phase for hosts to restore optimal bacterial communities [61]. On the other hand, the reduced beta diversity among nesting birds suggests that parenting behavior, including nest construction, may foster a more uniform gut microenvironment, perhaps due to shared physiological demands [62, 63]. The convergence could also indicate that nest construction behavior depends on inputs from specific dominant bacteria, such as metabolites or neurological interactions [64]. In return, nesting behavior may facilitate the transmission of the dominant bacteria among birds [24], creating a feedback loop between host behavior and microbial community composition [4].

### Shifts in Campylobacteraceae and Lactobacillaceae families mark the transition to nesting status

We observed substantial shifts in two dominant bacterial families—*Campylobacteraceae* and *Lactobacillaceae*—within the gut microbiota of zebra finches across the treatment groups. Notably, *Campylobacteraceae* became prevalent in nesting zebra finches, consistent with our previous finding that adult zebra finches harbor abundant *Campylobacteraceae* population that can be transferred to their offspring via nests [24]. *Campylobacter* and related genera in the family *Campylobacteraceae* are oral and intestinal commensals in vertebrates [65], and they are abundant in healthy zebra finches [24, 26, 66, 67] and other pet songbirds [61].

The increased abundance of *Campylobacteraceae* in nesting finches may be linked to reduced immunocompetence during reproduction. Energetically expensive activities, such as breeding, can reduce body condition in zebra finches, potentially compromising their immune defense against potential pathogens [68, 69]. This mechanism corresponds to observations in chickens (*Gallus gallus*), where inefficient immune function causes persistent *Campylobacter* colonization [70, 71]. *C. jejuni* is one of the most common causes of gastroenteritis in wild and domestic animals [67, 72] although zebra finches are generally not affected. Regardless of the effect of *Campylobacteraceae* on zebra finches’ health, nesting behavior may enhance transmission between parents and offspring, further supporting their dominance.

In contrast, *Lactobacillaceae* abundance modestly reduced as zebra finches transitioned to nesting status. While *Lactobacillaceae* bacteria are known to improve immune function, metabolism and social behavior across various species [7, 12, 73,75], their reduced abundance in nesting zebra finches supports the observed declined body condition during reproduction [68, 69]. However, some *Lactobacillus* species can produce toxic metabolites or undesired amines [75], which might obstruct the initiation of nesting status. Thus, we are still uncertain whether the lower *Lactobacillaceae* abundance is the driver or consequence of nesting status.

The opposite trends in *Campylobacteraceae* and *Lactobacillaceae* abundance may also reflect their antagonistic relationship, previously observed in chickens [76]. *Lactobacillaceae* can compete with *Campylobacteraceae* for intestinal adhesion sites and induce mucosal immune responses against them [76]. Thus, either increased *Campylobacteraceae* may outcompete *Lactobacillaceae* in nesting finches, or reduced *Lactobacillaceae* may enable *Campylobacteraceae* growth. These functional interactions may even extend to other bacterial taxa, collectively influencing microbial community dynamics in birds during the transition to nesting status.

### Gut microbiota functional profiles reflect elevated nutritional and energetic demands in nesting zebra finches

Our analysis of gut microbiota functional profiles offers insights into how bacteria support host nesting behavior through metabolic interactions. Gut bacteria can synthesize and supply essential amino acids and vitamins to their hosts [77, 78]. Notably, the gut microbiota of nesting zebra finches showed elevated synthesis of essential amino acids (e.g., aromatic and branched-chain amino acids) and vitamins, potentially meeting the increased nutritional demands associated with breeding behavior, including nesting behavior [79, 80]. The findings align with research across varying species, indicating the importance of essential amino acids in nesting and caregiving behaviors. For instance, depletion of dietary tryptophan reduces nesting behavior in mice, whereas supplementation enhances it [81]. Similarly, buff-tailed bumblebees (*Bombus terrestris*) exhibit delayed nest initiation when fed pollen with low essential amino acid content [82].

The gut microbiome of nesting zebra finches also exhibited an enriched profile of energy metabolism pathways—the citrate cycle and biosynthesis of pantothenate and Coenzyme A. This enrichment possibly reflects the heightened energy demands of nesting behaviors. Energy-demanding behaviors can alter host metabolism, changing the nutrient environment of gut microbes and forcing them to adapt their metabolic processes. In addition, increased bacterial ATP production may suppress protective IgA generation against enteropathogens [83], potentially explaining the increased *Campylobacteraceae* (potential pathogens) abundance in nesting birds. These findings highlight the bidirectional nature of host-microbe interactions.

The enriched functional pathways also suggest psychological stress in nesting finches. Nesting finches showed enriched synthesis of branched-chain amino acids and pantothenate, while non-nesting birds showed elevated synthesis of ornithine (Fig. S4, Table S5). Similar intestinal metabolic patterns in mammals correlate with depression and autism spectrum disorder [84, 85], suggesting that finch nesting behavior may be associated with elevated stress levels. This is consistent with observations of elevated stress levels in northern gannets (*Morus bassanus*) during the incubating and early chick-rearing periods [86] and reduced body condition in breeding zebra finches [68, 69]. *Lactobacillus* bacteria can produce ornithine from arginine to promote healthy gut mucosa formation [87], which helps prevent anxiety and depression caused by leaky gut and chronic inflammation [88]. The higher *Lactobacillus* abundance and enriched ornithine synthesis in the gut microbes of non-nesting birds suggest that nesting demands may impact both physical and psychological well-being through the microbiota-gut-brain axis.

### Sex-specific changes in gut microbiome during transition to nesting status

Female zebra finches showed more pronounced changes than males in gut microbiome diversity (Figs. S1 and 2), dominant taxa (Fig. S3) and functional potentials (Fig. S4) when entering nesting status. These sex-specific differences likely reflect distinct physiological demands during reproduction, particularly egg production, which may necessitate specific microbial consortia to optimize energy extraction and nutrient utilization. While zebra finches are opportunistic breeders maintaining year-round reproductive readiness, females and males differ in their gonadal preparation [28, 89]. Females maintain ovarian follicles at a medium-developed stage, requiring about two weeks to become fully yolked when suitable breeding conditions arise. In contrast, males maintain testes at a more advanced stage during non-breeding periods, allowing faster activation of reproductive function [28, 89]. The stronger shift of gut microbiota in females upon entering nesting status likely reflect these sex-specific differences in gonadal development.

Our results contrast with Maraci et al. [26], which reported decreased microbial alpha diversity in male zebra finches but not females during parental care. This discrepancy could be attributed to difference in sampling timing. While Maraci et al. [26] examined later breeding stages from incubation to chick-rearing and fledgling, our study focused on the nest construction stage. This early stage provides novel insights into parenting behaviors that occur when individuals anticipate offspring, rather than responding to their presence. We demonstrate that female zebra finches, but not males, exhibit dramatic changes in gut microbial community after exposure to breeding conditions—access to both mates and nest materials.

### Dichotomic roles of gut microbiome in initiating nesting status versus maintaining nesting actions

The sexual difference in nesting actions and gut microbiome changes prompted us to examine the relationship between microbial characteristics and specific nesting movements in zebra finches. Within the nesting group, a positive correlation between Shannon index and nest construction latency indicates that higher gut microbial diversity may delay the onset of nesting behavior. This result is consistent with our inter-group analysis showing that low microbial diversity characterizes the transition to nesting status. In contrast, Faith’s PD showed a significant positive correlation with female presence in nest boxes, suggesting that microbial diversity may show differential interactions with nesting initiation versus ongoing nesting actions, particularly in females.

Some individual bacterial taxa also showed diverging relationships with nesting phase transitions and specific nesting actions. In the nesting group, higher *Lactobacillaceae* abundance correlated with delayed nesting onset but, paradoxically, positively associated with male-specific nesting action—fetching nest materials—once nesting status commenced. Similarly, the abundance of *Lactobacillus aviarius* within the *Lactobacillaceae* family positively correlated with time spent in nest boxes by females and both sexes combined. In contrast, the *Campylobacteraceae* family and its one unnamed species (*Campylobacter sp.*) were linked to shorter nest-construction latency, consistent with their increased abundance in nesting compared to non-nesting birds.

While *Campylobacteraceae* emerged as the predominant family of gut microbiota in nesting birds, *Lactobacillaceae* maintained significant correlations with ongoing nesting actions, highlighting its contribution to the practice of nest construction. The contrasting effects of *Lactobacillaceae* and microbiome diversity across nesting stages and between sexes warrant further investigation to how gut bacteria influence complex behavioral phenotypes through modulation of brain gene expression.

### Links between gut microbiome diversity, taxa, and brain gene expression related to nesting behavior

Our analysis revealed correlations between gut microbiome characteristics and the expression of social hormone and receptor genes in the nesting-associated brain regions of zebra finches. Specifically, the expression of *vipr1*,* vip*,* avpi1* and *drd3* in SBN, AMP and PM positively correlated with gut microbiome diversity and *Lactobacillaceae* family abundance, including *Lactobacillus aviarius* and *L. intermedius*. Moreover, *vip* was previously identified as a differentially expressed gene between nesting and non-nesting zebra finches [32]; Table S3). Our findings build upon previous research demonstrating the role of social hormones like vasopressin and dopamine in zebra finch nesting behaviors [33, 90, 37], suggesting that gut microbiome diversity and *Lactobacillaceae* bacteria may interact with nesting behavior through neurohormonal modulation. This mechanism parallels existing research showing how gut microbes can affect neural function. For example, a study found that *Lactobacillus* strain consumption changed GABA receptor mRNA expression in the mouse brain via the vagus nerve, reducing anxiety-related behavior [12].

We also revealed correlations between the expression of genes in the GnRH system and gut microbiome diversity. Furthermore, Fang et al. [33] showed that *gnrh1* and *ptpn5* were differentially expressed between nesting and non-nesting zebra finches (Table S3), implying the links between the GnRH system, gut microbiome composition, and nest construction behavior. The GnRH neuronal system also modulates nest-building behavior in tilapia fish (*Oreochromis niloticus*; [55]). In addition, in response to GnRH binding to GnRHR, PTPN5 promotes follicle-stimulating hormone secretion in the pituitary gland of mice [54]. Studies have found that sex hormones and gonadal development regulate nesting behavior in birds [51–53]. In zebra finches, correlations between gut microbial diversity and the expression of *ptpn5*, *gnrh1* and *gnrhr* genes in nesting-associated brain regions, especially in females, support a connection between nesting status and egg production. This explains why female zebra finches have stronger changes in gut microbiome when entering nesting status, with follicle maturation potentially regulated by gut microbiome-induced brain gene expression.

Our results also imply that the differential effects of gut microbiota across nesting stages and between sexes emerge from divergent brain gene expression patterns. Gut microbiome Faith’s PD showed contrasting correlations with *gnrhr* gene expression across brain regions: negative in the AMP region for all individuals, but positive in the SBN region for nesting individuals. In addition, *Lactobacillaceae* abundance exhibited sex-specific correlations—positively correlated with *avpi1* expression in female SBN while showing a negative correlation in male DNP (although the latter relationship became insignificant after multiple correction).

The observed correlations between gut microbiome characteristics and social hormone and follicle-related gene expression are consistent with a potential pathway of the microbiota-gut-brain axis that may influence avian nest-construction behavior. The findings suggest that the gut microbiome could contribute to the regulation of complex behaviors via brain gene expression. However, several limitations should be acknowledged. The modest sample sizes for brain gene expression analysis (five individuals per sex per treatment) constrain our ability to draw definitive conclusions about the interactions between gut microbiome, gene expression and nesting behavior. More critically, our correlative findings require validation through manipulative experiments to establish causality and determine whether the microbiota-gut-brain axis directly regulates nest-construction behavior in birds.

## Conclusions

Our study reveals complex relationships between the gut microbiome and nest-building behaviors in zebra finches, offering new perspectives on the biological underpinnings of host–microbe interactions. Female nesting birds exhibit convergent microbiota structure dominated by *Campylobacteraceae*, reflecting sex-specific physiological demands during nesting. Metabolic pathways analysis of the gut microbiome suggests elevated nutritional and energetic requirements especially in nesting females. Moreover, gut microbial diversity and *Lactobacillaceae* abundance correlate with brain gene expression, offering insights into the neurohormonal mechanism linking symbiotic bacteria to nest construction. Our findings illuminate how gut microbes contribute to adult brain plasticity, enabling behavioral adaptation to parenting demands. In return, nesting may promote gut microbiome transmission between hosts at parenting phases. These interactions shape the co-evolution between gut microbes and hosts. Future research should experimentally test whether *Campylobacter* and *Lactobacillus* species causally influence avian nesting behavior and uncover the precise mechanisms involved, thereby advancing our understanding of the microbiome-gut-brain axis in behavioral ecology.

## Supplementary Information

Below is the link to the electronic supplementary material.

Supplementary Material 1

Supplementary Material 2

## Data Availability

The sequence reads used in this study are deposited in the NCBI Sequence Read Archive under the BioProject PRJNA1115942.
