# Supplementary material for: Microbiota-gut-brain axis in avian parenting: gut microbiome associates with nest-construction behavior and neural gene expression in a songbird
Source: Anim Microbiome. 2025 Nov 18;7:120. doi: 10.1186/s42523-025-00486-w (PMC12625345; doi:10.1186/s42523-025-00486-w)
Supplement: Supplementary file 1 — Supplementary Material 1 [file 42523_2025_486_MOESM1_ESM.docx]

**Microbiota-gut-brain axis in avian parenting: gut microbiome associates with nest-construction behavior and neural gene expression in a songbird**

Cheng-Yu Chen^1,2^, Hao-Chih Kuo^2^, Yi-Ting Fang^2,3^, Chia-Wei Lu^2^, Shih-Kuo Chen^1^, and Chih-Ming Hung^2^*

^1^Department of Life Science, National Taiwan University, 10617 Taipei, Taiwan

^2^Biodiversity Research Center, Academia Sinica, 11529 Taipei, Taiwan

^3^Animal Behavior Graduate Group, University of California, Davis, Davis, CA 95616, USA

* Corresponding author. E-mail address: [cmhung@gate.sinica.edu.tw](mailto:cmhung@gate.sinica.edu.tw) (C.-M.H.)

**Supplementary Figures: S1 – S5**

**
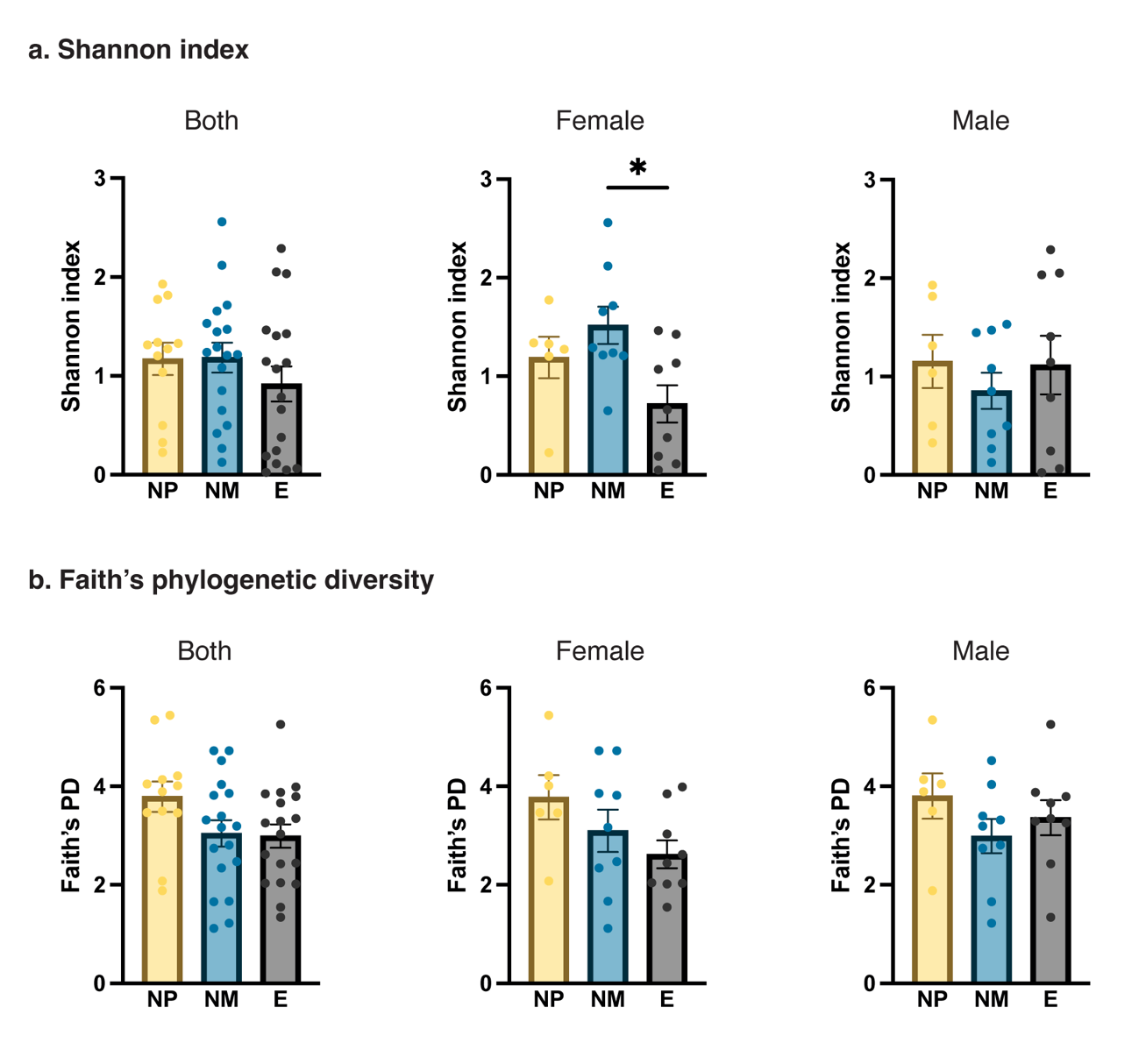
**

**Figure S1. Alpha diversity of gut microbial communities across different treatment groups.** We use rarefied data (15,000 reads per sample) to measure alpha diversity based on (a) Shannon index and (b) Faith’s phylogenetic diversity (Faith’s PD). The bar plots present means ± standard errors. Differences among three treatment groups are evaluated using non-parametric Kruskal-Wallis tests followed by Dunn’s post-hoc tests (*, *adj*. *p* < 0.05).

**
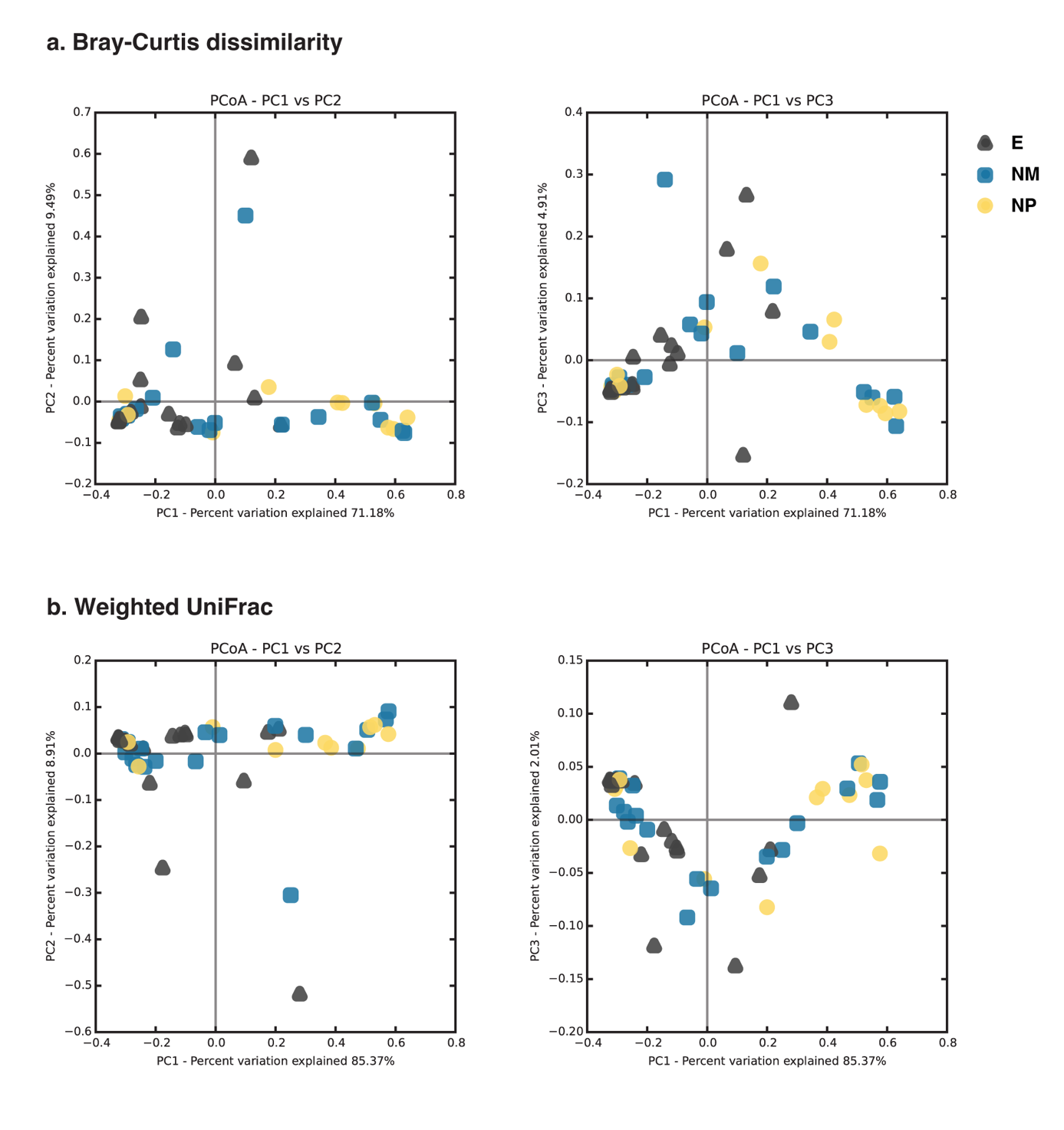
Figure S2. Principal coordinate analysis of gut microbial compositional variation among zebra finches from different treatment groups.**

Principal coordinal analysis is carried out based on (a) Bray-Curtis dissimilarity and (b) weighted UniFrac distance between samples using ASV relative abundances. Sample colors represent different treatments.


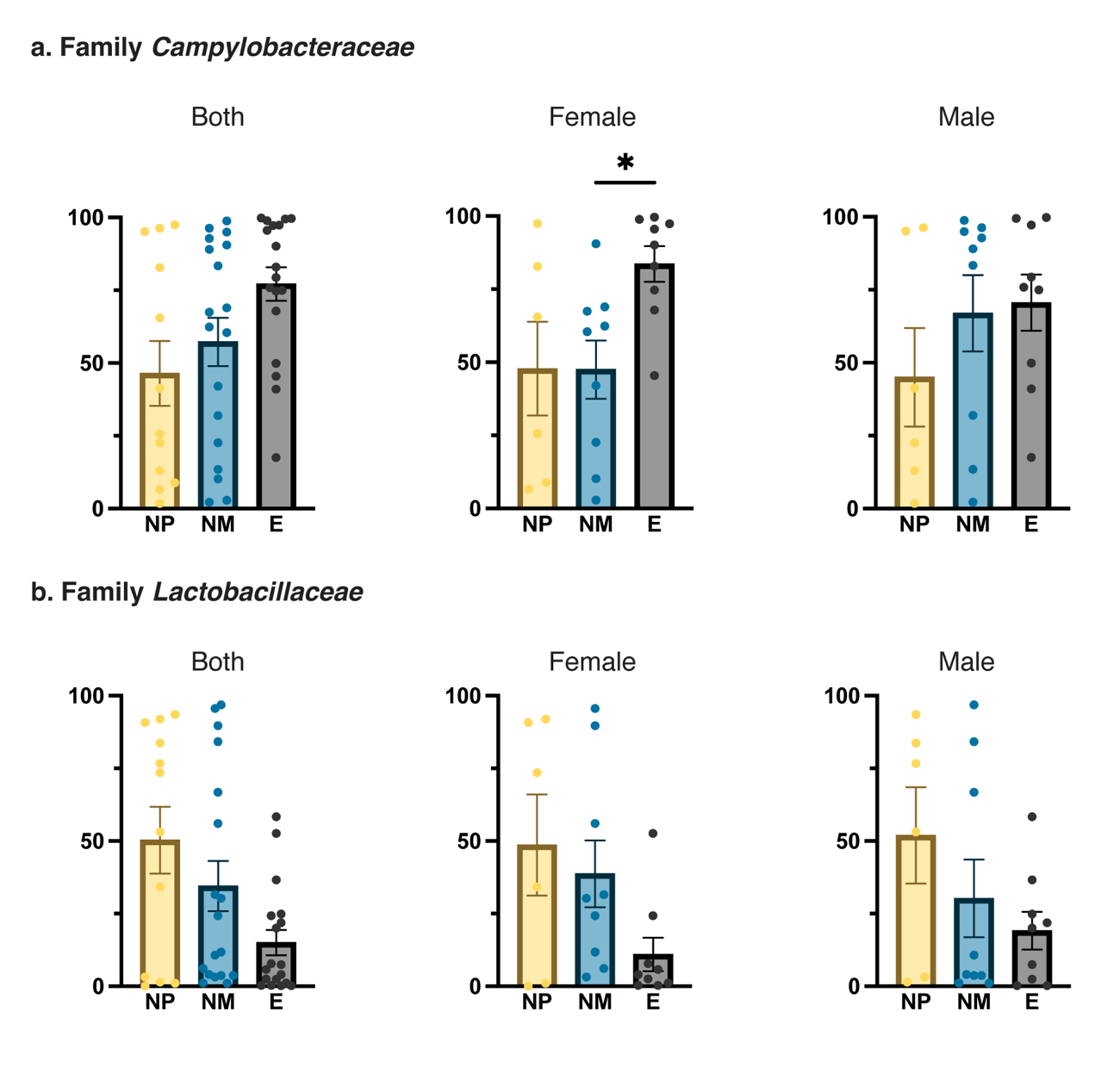


**Figure S3. Relative abundance of gut bacterial families (a) *Campylobacteraceae* and (b) *Lactobacillaceae* across different treatment groups.** Differences between treatment groups are evaluated using Kruskal-Wallis tests followed by Dunn’s post-hoc tests (* *adj*. *p* < 0.05).


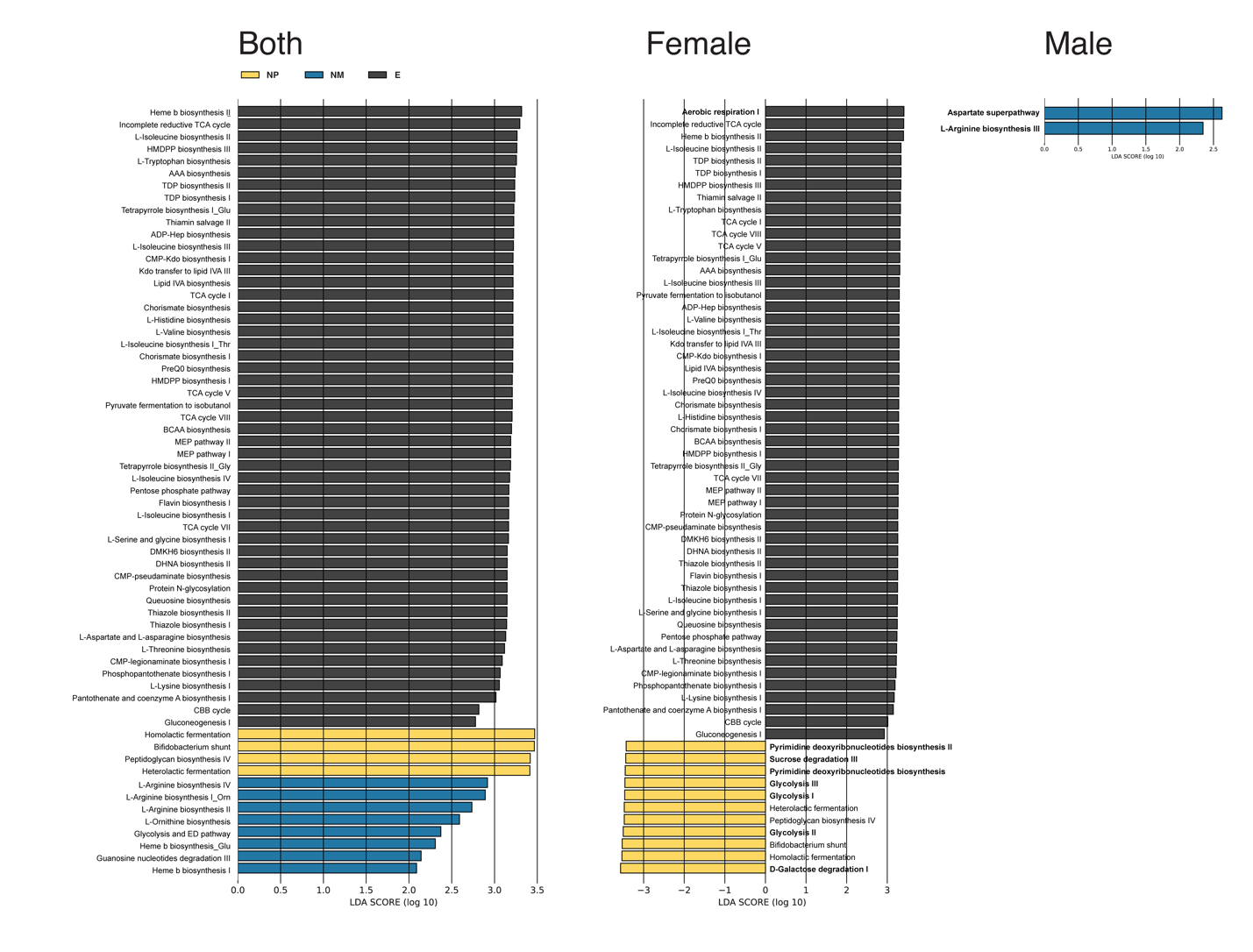


**Figure S4.** **Enrichment of gut microbiota-associated functional pathways across treatment groups.** PICRUSt2 analysis is conducted to sex-combined (corresponding to figure 4), female-specific and male-specific data, respectively. Significance of enrichment is evaluated using the linear discriminant analysis effect size (LefSe) under criteria of *p* < 0.05 and LDA score >2.

**Figure S5. Correlations between dominant gut bacterial family abundance and nesting actions.** Scatter plots depict relationships between the relative abundance of dominant gut microbial family and nesting actions in E-group zebra finches. Upper panels show sex-combined correlation between latency to initiate nesting and (a) *Lactobacillaceae* abundance as well as (b) *Campylobacteraceae* abundance (n = 9 pairs, blue points indicate males and grey points indicate females). Lower panels show male-specific correlation between the duration of fetching nest materials and (c) *Lactobacillaceae* abundance as well as (d) *Campylobacteraceae* abundance (n = 9 birds). Solid lines indicate linear relationship and shaded regions show 95% confidence intervals. Spearman’s rank correlation coefficient (ρ) and corresponding p-values are shown for each relationship.
